# Management of a Large Palatal Ulcer: Mucous Membrane Pemphigoid

**DOI:** 10.7759/cureus.35619

**Published:** 2023-02-28

**Authors:** Simran D Badki, Vidya Lohe, Vrushali B Zamare, Suwarna Dangore-Khasbage, Ravindra P Kadu, Mayur B Wanjari

**Affiliations:** 1 Oral Medicine and Radiology, Sharad Pawar Dental College and Hospital, Datta Meghe Institute of Higher Education and Research, Wardha, IND; 2 Oral Medicine and Radiology, Sharad Pawar Dental College and Hospital, Datta Meghe Institute of Medical Sciences, Wardha, IND; 3 Pathology, Jawaharlal Nehru Medical College, Datta Meghe Institute of Higher Education and Research, Wardha, IND; 4 Research and Development, Jawaharlal Nehru Medical College, Datta Meghe Institute of Higher Education and Research, Wardha, IND

**Keywords:** ulcer, mucous membrane, vesiculobullous, inflammatory, pemphigoid

## Abstract

Mucous membrane pemphigoid (MMP) is an autoimmune disorder that causes inflammatory changes and blistering of the subepithelial layer and is chronic and commonly related to the mucous membranes. It most commonly involves females in the fifth decade of life. In most of the cases, oral mucosa is involved. Dentists might be the first health professional to encounter and make a diagnosis of this rarely occurring disorder with mucocutaneous lesions. This article presents an MMP case report with clinical appearance, diagnosis, management, and follow-up.

## Introduction

Ulcerative and vesiculobullous lesions in the oral cavity have a similar clinical appearance, so differentiating one condition from another is challenging for oral physicians. Mucous membrane pemphigoid (MMP), bullous pemphigoid, linear IgA disease, erythema multiforme, dermatitis herpetiformis, and epidermolysis bullosa are all subepithelial vesiculobullous disorders. This disease primarily affects patients twice as females above 50 [1,2]. This disease presents with blistering and ulceration with erosions [3]. In MMP, the autoantibodies are directed in opposition to complement (C3)-associated proteins in the basement membrane, which is responsible for subepithelial split and subsequent formation of vesicles [4]. These antigens are in one of the basement membrane layers known as lamina lucida; however, the lamina densa can be associated in some cases. Subsets of MMP were diagnosed with the "salt-split skin" technique [2]. When discussing the incidence of MMP, only 2-10 people out of 100,000 individuals are involved [5].

MMP is less severe when compared with pemphigus [6,7]. The sites of involvement are all the mucosal surfaces like the oral, ocular, and oropharyngeal mucosa with the larynx and sometimes involving the genital region. In oral cavity vesicles, erosions are covered with pseudo membrane, desquamative gingivitis, and ulcers. Skin involvement is limited to the head, neck, and upper limbs [2,8]. After erosions, the ulcer heals with scarring, a unique feature of MMP. The cicatricial name pemphigoid is due to its scarring process [8]. MMP is associated with complications such as loss of vision, dysphagia, stenosis of the larynx, and anal or urethral strictures [2]. In the present case, only oral mucosal involvement was seen without any skin or genital lesions.

## Case presentation

A 59-year-old female reported fluid-filled blisters in the mouth for six months which burst to form painful ulcers leading to peeling of the mucosal surface of the palate in the last month. The lesion was associated with pain and a burning sensation while consuming hot and spicy foods. She visited a general physician 15 days back, where the antiseptic gel was prescribed for topical application on the lesion, but she did not get relief; therefore, she visited this hospital. The patient gave no history of trauma from hard food or any other mechanical insult to the palate. The patient had a history of a similar episode almost a year back at that time, the biopsy was done, and the patient was carrying the reports with her which stated subepithelial blistering and diagnosis of MMP.

**Figure 1:**
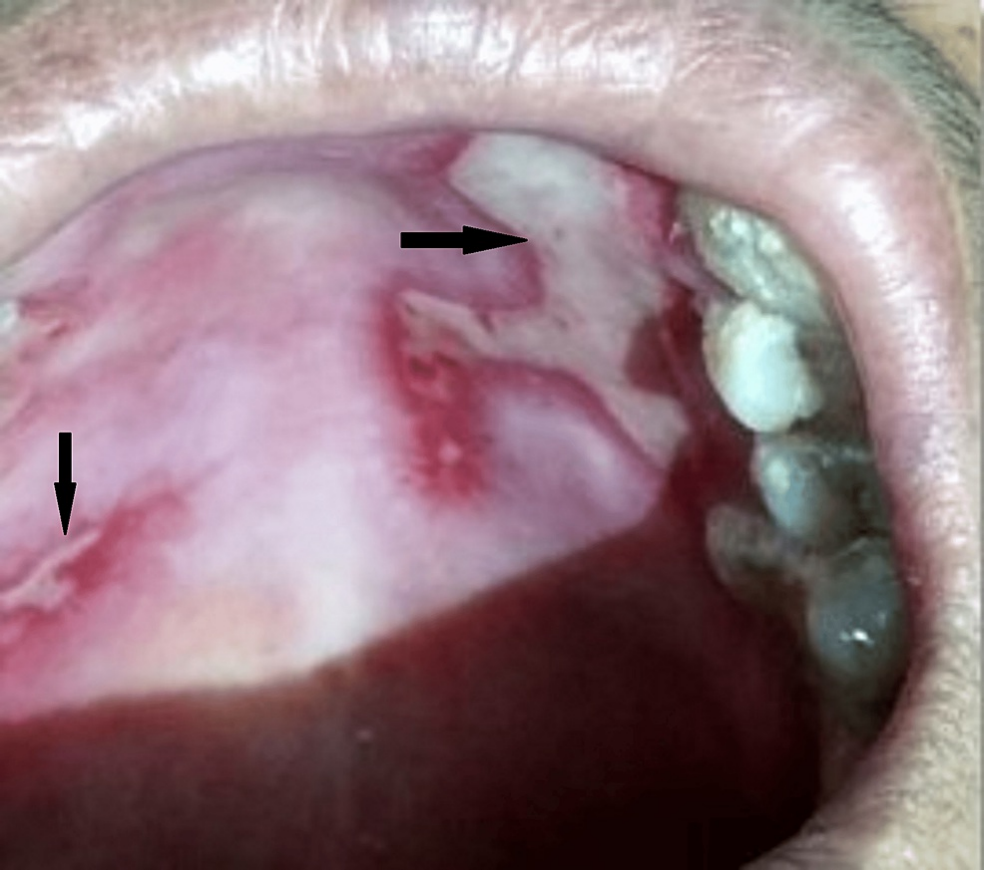
Two large ulcers covered with slough and surrounding erythema present on the hard palate.

Extra oral examination revealed no gross facial asymmetry, regional lymphadenopathy, and no involvement of ocular, cutaneous, or genital mucosa. Intraoral examination revealed two large ulcers localized on palatal mucosa with surrounding erythematous halo in the 13-16 region and 23-26 regions and surrounded by diffuse irregular borders covered with a pseudo membrane (Figure 1).

**Figure 2:**
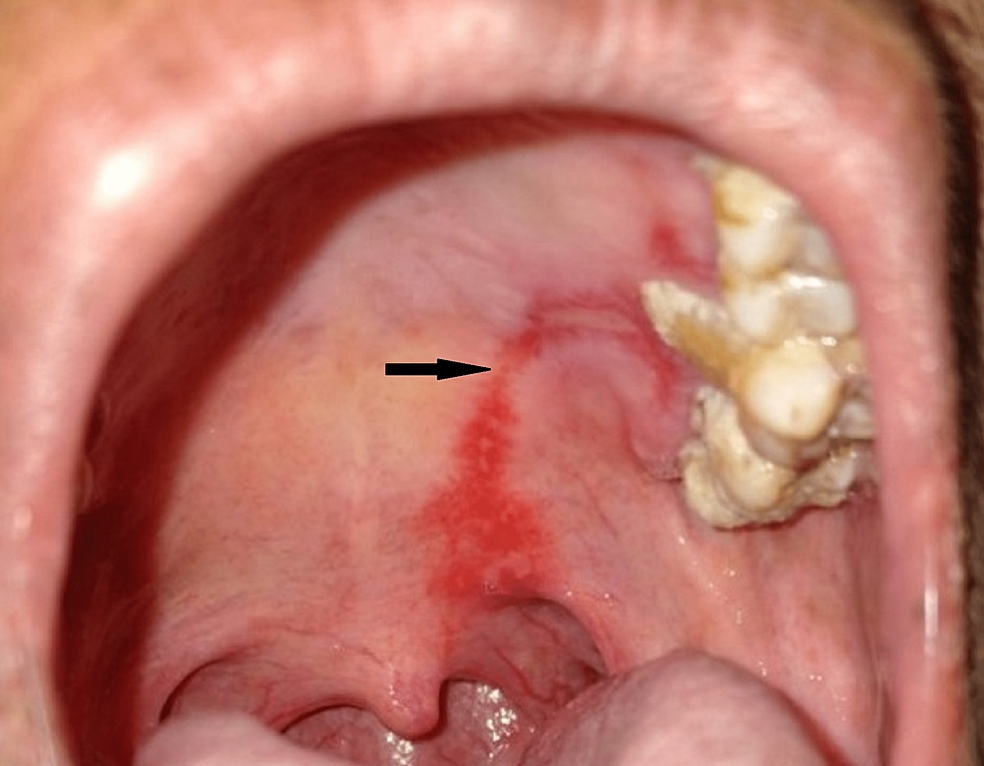
On the first follow-up visit after two weeks, the ulcers were healed by 70-80%.

The teeth in the vicinity showed plenty of plaque and calculus deposition. Severe gingival recession was associated with a palatal root of 26 and missing 16. Generalized gingival recession and gingival inflammation were present. The past biopsy results and clinical examination of the present lesion gave a provisional diagnosis of a subepithelial bullous lesion-like MMP. Triamcinolone acetonide gel was prescribed three times a day for seven days for local application on the lesion. She was asked to let the gel remain there for about 15-20 minutes, after which she was advised to rinse her mouth with water. The patient was kept on vitamin supplements once daily for 30 days. For the initial 30 days, the patient was recalled and reviewed every 14 days. The lesions healed significantly with topical steroids within two weeks of the treatment. (Figure 2).

**Figure 3:**
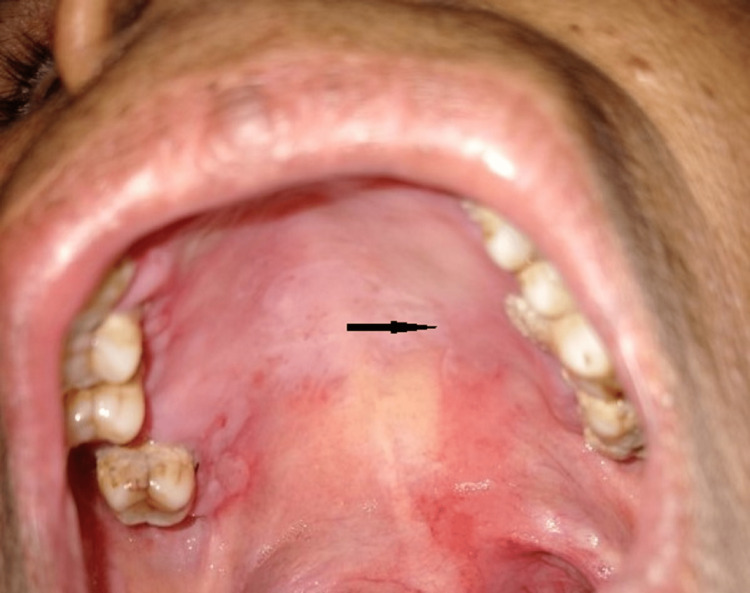
In the second follow-up visit, the ulcer on the hard palate showed complete healing without scarring.

The frequency of topical application of steroids was then reduced during the duration. However, the lesion started exacerbating again, so systemic steroid prednisone 10 mg/day once a day in the morning for five days was prescribed, and after five days, the dose was subsequently tapered to 5 mg till the attainment of maintenance dose without any adverse effects of the drug when treatment was going on. Finally, after two months, almost complete healing was seen on review (Figure 3), and follow-up was scheduled routinely.

## Discussion

The clinical heterogeneity of MMP and challenging diagnosis often lead to a substantial diagnostic delay and suboptimal treatment [3]. The prime lesion of MMP appears when autoantibodies directed against complement (C3)-associated proteins located in the lamina lucida, a basement membrane zone, cause a subepithelial split resulting in loss of adhesion of cell and subsequent vesicle formation [3,9]. Vesicles or bullae of MMP can involve any mucosal surface, but more than 80% of cases involve oral mucosa, and scarring is rare. In the present case, as the bullae were thick-walled, they remained intact for around 4 to 5 months. The breaking of bullae or vesicles leads to irregular erosive lesions with yellowish slough with surrounding erythema. The erosions are more self-limiting and spread more slowly than pemphigus. Various sites can be involved, such as the tongue, hard palate, soft palate, buccal or labial mucosa, and alveolar ridge [8]. In this case, there was the involvement of the palate. The second most common site is conjunctiva; the conjunctival lesions can lead to symblepharon formation after scar formation. Corneal damage leads to blindness. Lesions on the genital mucosa are painful and can lead to sexual dysfunction. However, the present case showed no genital or eye involvement. Laryngeal involvement shows symptoms like hoarseness of voice, pain in the larynx, and difficulty in breathing, and it can also lead to death due to asphyxiation. Involvement of the esophagus may cause dysphagia, which can further lead to debilitation and death [3]. The most important clinical sign in MMP is desquamative gingivitis. Loss of stippling, erythematous gingiva, and extending apically from gingival margins to alveolar mucosae are essential clinical features. They could be mild, small patches to widespread erythema with a glazed appearance [10,11]. In the present case, generalized erythematous gingiva was seen; this can be attributed to poor oral hygiene on the part of the patient due to the painful condition she was suffering from.

However, for the diagnosis of MMP, no consensus reference standard has been established. The first International Consensus stated clinical criteria and direct immunofluorescence microscopy are enough for establishing a diagnosis of MMP [12]. A biopsy is generally not advised in gingival lesions, as chronic inflammation can complicate the diagnosis [8]. If the preferred site for a biopsy is a vesicle or tissue around the lesion and not an erosion, it will show only loss of epithelium. Subepithelial split with variable infiltrates of inflammatory cells which contain eosinophils in lamina propria are considered characteristic histopathological features [12,13]. Taking the provisional diagnosis into consideration, the present case was managed by topical and systemic steroids and the patient responded satisfactorily to the same; hence biopsy of the lesion was not carried out.

Good oral hygiene should be taught to the patient, including cleaning of teeth twice daily using a soft toothbrush and toothpaste, daily flossing, and regular dental checkup and scaling every 3-6 months [14,15].

When systemic steroids show no significant response, then along with steroids, azathioprine, cyclophosphamide, dapsone, and mycophenolate mofetil are used as an adjunct therapy and have proven effective in treating MMP. Intravenous immunoglobulin, rituximab, and infliximab are effective in reducing autoantibody production and hence cause a decrease in inflammation. Other measures, such as cryotherapy or low-energy laser phototherapy, can be used in some cases [16].

## Conclusions

MMP does not end and is often associated with the rise and fall of clinical signs and symptoms. Early and accurate diagnosis can lead to satisfactory management of the condition and prevent further areas of involvement. Physicians should use pathologic and immunological techniques to help diagnose such patients. Appropriate adjuvant systemic immunosuppressive therapy is required in patients with chronic disease. Besides advances in available anti-inflammatory drugs and biologics, cuts are a significant problem in many cases. Surgical intervention is not advisable; however, restoring function and improving health quality may be necessary. The role of the dentist is prime in early diagnosis. Standard and regular follow-ups must be done to prevent escalation and remission.

## References

[1] Chan LS, Fine JD, Briggaman RA, Woodley DT, Hammerberg C, Drugge RJ, Cooper KD (1993). Identification and partial characterization of a novel 105-kDalton lower lamina lucida autoantigen associated with a novel immune-mediated subepidermal blistering disease. J Invest Dermatol.

[2] Xu HH, Werth VP, Parisi E, Sollecito TP (2013). Mucous membrane pemphigoid. Dent Clin North Am.

[3] Greenberg M, Glick M (2015). Burket's Oral Medicine Diagnosis and Treatment. https://rlmc.edu.pk/themes/images/gallery/library/books/dental/%5BLester_William_Burket,_Martin_S._Greenberg,_Micha(BookFi).pdf.

[4] Dharman S, Muthukrishnan A (2016). Oral mucous membrane pemphigoid - two case reports with varied clinical presentation. J Indian Soc Periodontol.

[5] Dear JW, Lilitkarntakul P, Webb DJ (2006). Are rare diseases still orphans or happily adopted? The challenges of developing and using orphan medicinal products. Br J Clin Pharmacol.

[6] Scully C, Carrozzo M, Gandolfo S, Puiatti P, Monteil R (1999). Update on mucous membrane pemphigoid: a heterogeneous immune-mediated subepithelial blistering entity. Oral Surg Oral Med Oral Pathol Oral Radiol Endod..

[7] Bruch-Gerharz D, Hertl M, Ruzicka T (2007). Mucous membrane pemphigoid: clinical aspects, immunopathological features and therapy. Eur J Dermatol.

[8] Schmidt E, Zillikens D (2013). Pemphigoid diseases. Lancet.

[9] Scully C, Lo Muzio L (2008). Oral mucosal diseases: mucous membrane pemphigoid. Br J Oral Maxillofac Surg.

[10] Bagan J, Lo Muzio L, Scully C (2005). Mucosal disease series. Number III. Mucous membrane pemphigoid. Oral Dis.

[11] Silverman S, Gorsky M, Lozada-Nur F, Liu A (1986). Oral mucous membrane pemphigoid. A study of sixty-five patients. Oral Surg Oral Med Oral Pathol.

[12] Sami N, Bhol KC, Ahmed AR (2002). Treatment of oral pemphigoid with intravenous immunoglobulin as monotherapy. Long-term follow-up: influence of treatment on antibody titres to human alpha6 integrin. Clin Exp Immunol.

[13] Carrozzo M, Dametto E, Fasano ME, Broccoletti R, Carbone M, Rendine S, Amoroso A (2014). Interleukin-4RA gene polymorphism is associated with oral mucous membrane pemphigoid. Oral Dis.

[14] Calabresi V, Carrozzo M, Cozzani E (2007). Oral pemphigoid autoantibodies preferentially target BP180 ectodomain. Clin Immunol.

[15] Knudson RM, Kalaaji AN, Bruce AJ (2010). The management of mucous membrane pemphigoid and pemphigus. Dermatol Ther.

[16] Di Zenzo G, Carrozzo M, Chan LS (2014). Urban legend series: mucous membrane pemphigoid. Oral Dis.

